# Persistent orocutaneous and anal fistulae induced by nicorandil: a case report

**DOI:** 10.1186/1752-1947-3-119

**Published:** 2009-11-12

**Authors:** Cyndi Goh, Sally CY Wong, Colin Borland

**Affiliations:** 1 Department of Medicine, Hinchingbrooke Hospital, Hinchingbrooke Heath Care NHS Trust, Huntingdon, Cambridgeshire PE29 6NT, UK

## Abstract

**Introduction:**

Although nicorandil is prescribed widely, awareness of its potential to cause serious complications to the gastrointestinal tract mucosa is limited. Whilst nicorandil-induced oral and anal ulceration is well documented in the literature, nicorandil-induced fistulation is not. This is the first report in the literature of a single patient demonstrating simultaneous orocutaneous and anal fistulae during nicorandil therapy. Two separate cases of orocutaneous and anal fistulae associated nicorandil usage have previously been documented in specialist journals.

**Case presentation:**

A 71-year-old Caucasian man presented with a 3-year history of concurrent orocutaneous and anal fistulae. He had been exposed to 30 mg twice-daily nicorandil therapy for 4 years. Both fistulae responded poorly to intensive and prolonged conventional treatment but healed promptly on reduction and eventual withdrawal of nicorandil therapy.

**Conclusion:**

Management of resistant cases of orocutaneous and anal fistulae in patients on high-dose nicorandil therapy may be impossible without reduction or even withdrawal of nicorandil.

## Introduction

Nicorandil was first introduced as an anti-anginaltreatment in Japan over 20 years ago and is widely prescribed in the UK [1]. As a potassium channel opener with a nitrate component, nicorandil reduces both preload and afterload [2], and its use is often employed in the difficult challenge of managing patients with refractory or unstable angina pharmacologically. It is recommended as second-line therapy in reducing the symptoms and signs of angina pectoris and myocardial ischaemia[3]. Nicorandil is generally well tolerated with common side effects including flushing, headache and nausea [4]. However, it has become increasingly recognised that its usage is also associated with debilitating ulcer formation. Since the first French report of nicorandil-induced oral ulcers in 1997 [5], there have been reports of other nicorandil-induced ulcers, including anal, peri-anal [6,7], vulval and peristomal ulcers [8]. A single case of small and large bowel ulcers leading to a perforated terminal ileum has also been described in the literature [9].

We describe a single case involving both orocutaneous and anal fistulae in a patient on nicorandil. Unlike its well-known association with ulcer formation, reports of nicorandil-induced fistulae formation are very rare in the literature. To our knowledge, only a single paper [8] has been published describing four cases of fistulating large bowel disease, one 'letter to the editor' [10] describing a case of orocutaneous fistula following nicorandil therapy and another letter [11] reporting a case of nicorandil-associated peri-anal fistula formation.

Many of these case reports appear in specialised cardiology, dermatology, surgery and dentistry journals. Therefore, given the widespread prescribing of nicorandil, this case report aims to generate increasing awareness of its serious complications amongst general physicians.

## Case presentation

A 71-year-old Caucasian man was admitted to our hospital under the general medical team in July 2007 with a 15-month history of recurrent cellulitis over the right maxillary region of his face. There were no obvious aetiological factors apart from stable tablet-controlled type 2 diabetes mellitus. Wound swabs for microscopy, culture and sensitivity were repeatedly negative and there was no history of trauma or underlying dermatological disease. The patient had suffered from a dental abscess involving the right upper quadrant molars, but this was noted only 13 months following his initial presentation in 2006; tooth extractions performed with a view to curing his facial cellulitis had limited effect on this. Multiple courses of broad-spectrum oral and intravenous antibiotics had been ineffective in treating the cellulitis. Two computed tomography (CT) scans of the paranasal sinuses performed in February and July 2007 had ruled out sinusitis as an underlying cause. The patient had declined to have a skin biopsy.

Several days after admission, the patient developed an abscess over the right maxillary region and began complaining of a foul taste in his mouth from the discharging abscess. It was at this point that a discharging fistula between the right nasolabial fold and the right buccal sulcus was diagnosed clinically. Following reviews by clinicians in the areas of dermatology, microbiology, ENT (ear nose and throat) and maxillofacial surgery, the significance of this patient's nicorandil treatment (30 mg twice daily) initiated 4 years previously, was recognised. His drug history also included spironolactone, isosorbide mononitrate, warfarin, gliclazide, bisoprolol, paroxetine and furosemide. On direct questioning, the patient admitted to having experienced small oral ulcers involving his tongue 6 months preceding his admission, although he had not sought medical advice as they had not been particularly troubling. On this occasion, he had no oral ulcers.

On discussion with the cardiologists, the patient was put on a 4-week long tapering regimen of nicorandil resulting in complete withdrawal of nicorandil therapy. There was some clinical improvement noted at 2 weeks into this reducing regime and a month after his nicorandil therapy had ceased, his fistula had healed and the cellulitis disappeared completely for the first time in 15 months.

A retrospective review of his imaging confirmed a fistula in the right maxillary region on the CT scan performed in February 2007. The patient had not complained of any symptoms suggestive of a fistula at this point. However, retrospective questioning revealed that he had had symptoms of a 'foul taste' in his mouth during this time, which he had initially attributed to the dental abscess.

In addition, investigation of the patient's case notes revealed that he had had a persistent peri-anal fistula over a period of 1 year from 2004 to 2005. This low anal fistula was initially laid open surgically in 2004. However, this procedure was complicated by poor wound healing and abscess formation requiring two further surgical procedures over the next year. The fistula finally healed in mid-2005, corresponding to a coincidental nicorandil dose reduction from a total daily dose of 60 mg to 30 mg during the preceding month.

## Discussion

More than 50 cases of nicorandil-induced oral ulcers have been reported. A prospective study by Marquart-Elbaz *et al*. [12] reported a 5% prevalence of oral ulceration in patients on nicorandil, compared with patients on various other anti-anginal medications. In contrast, a prescription event monitoring study assessing 13,260 patients reported a much lower incidence of 0.4% [13]. The median onset of ulcer formation following commencement of nicorandil has been found to be 12 months, with a range of 2-36 months [14]. These ulcers are large (up to several centimetres is size), persistent, recurring, deep and painful but healpromptly after withdrawal of nicorandil [15]. The tongue is often affected, but the ulceration can also involve the hard palate, buccal and labial mucosa [14,16]. The lesions have a consistent appearance from patient to patient, and were initially described as 'aphthous ulcers'. However, a recent study in 2003 involving 60 dermatologists reported the ulcers as 'non-aphthous' in nature [17] (Table 1).

**Table 1:** A comparison of aphthous and non-aphthous ulcers

Aphthous ulcer	Non-aphthous ulcer
Often has yellow base Erythematous halo	Punched out appearance No surrounding erythematous halo

Biopsies of these ulcers show non-specific features of ulceration [15], that is, granulation tissue associated with acute inflammation. These ulcers show a poor response to topical steroids [14,15]. Transient healing is seen in some cases treated with colchicine or thalidomide but the ulcers relapse rapidly once treatment is stopped [14]. The definitive treatment is withdrawal of nicorandil, which must be done with caution, instituting a gradually tapering regimen and starting another anti-anginal if necessary, to avoid exacerbation of anginal symptoms. The Medicines and Healthcare Products Regulatory Agency advises that nicorandil withdrawal be carried out only under the supervision of a cardiologist [18]. Patients often describe a dramatic decrease in pain within 2 weeks of withdrawal, and healing in around 4 weeks, mostly without scarring [16].

A single case of orocutaneous fistula has been described in the form of a 'letter to the editor' in 2005 [10]. The patient described had a full thickness defect of his left cheek and a deeply ulcerated area in the floor of his mouth. Nicorandil was stopped and nasogastric feeding was commenced to aid healing inside the mouth. The mouth ulcer healed in 6 weeks but a facial flap was required to close the fistula. The patient recovered well with no recurrence of the oral ulcer.

These ulcers and fistulae are distressing for patients with resulting dysphagia, weight loss and occasionally depression [12]. In cases where the diagnosis is in doubt, biopsy should be performed to rule out other aetiologies, especially malignancy. Super-infection caused by bacteria or fungi may also need to be excluded. The significance of this was demonstrated in a recent case report of a patient with nicorandil-induced oral ulceration and cervicofacial actinomycosis [19].

The phenomenon of anal ulceration was first recognised by Watson *et al*. [6] and more cases have been reported since. Patients often complain of anal pain, pain on defecation or bleeding per rectum and can present with acute anal fissures or suspicious-looking anal ulcers. These ulcers are, again, deep punched out cavities with well-circumscribed edges. Histological examination of these areas shows non-specific inflammation with no evidence of malignancy [6,7]. Similarly to the oral ulcers, the anal and peri-anal ulcers demonstrate dramatic improvement following withdrawal of nicorandil in terms of pain and appearance [6-8]. However, in contrast to oral ulcers, complete healing of ulcers in the gastrointestinal tract takes longer, ranging from 4 to 10 months following nicorandil withdrawal in one study [8].

Kamath *et al*. [11] described a single case of a patient presenting with a low peri-anal fistula that failed to respond to routine surgical management. The fistula was surgically laid open on two occasions over an 18-month period. However, the postoperative healing was complicated both times by development of a large, non-healing ulcer. Therefore, the patient underwent an abdominoperineal excision with defunctioning colostomy. Despite this, the peri-anal ulcer persisted and new ulcers began to form on the skin of the scrotum and groin. Six weeks after stopping his 20 mg twice daily dose of nicorandil, the scrotal ulcer healed. At 12 weeks, the peri-anal ulcer had also healed completely.

## Pathogenesis

The pathogenesis of nicorandil-induced ulceration is unclear. Some have postulated a local effect of nicorandil metabolites, as the drug is known to concentrate in the saliva. This however, does not explain its association with anal ulcers, as nicorandil is mainly excreted in urine. A 'vascular steal phenomenon' has also been proposed although neither the pharynx nor the anus are areas of vascular watershed [6]. Some authors have advocated a dose-dependent relationship as most patients with nicorandil-induced ulcers are on a dose of at least 20 mg twice a day [14-16] but the phenomenon has been reported even with doses of 10 mg daily [20].

More recently, Trechot *et al*. [21] hypothesised that prolonged therapy with nicorandil may result in its metabolites (nicotinamide and nicotinic acid) merging with and saturating the endogenous pool of nicotinamide adenine dinucleotide (NAD)/nicotinamide adenine diphosphate (NADP). This accumulation is thought to allow nicotinic acid to become abnormally distributed in areas such as the mucosa or skin, leaving it susceptible to ulceration.

## Conclusion

Despite uncertain knowledge of the mechanisms involved, evidence for the association between nicorandil and gastrointestinal tract ulceration is strong, with rapid resolution after its withdrawal. Therefore, it is important to consider nicorandil as part of the differential diagnosis in patients with persistent or multiple ulcers and/or fistulae of the gastrointestinal tract to avoid unnecessary surgical intervention amongst a group of high-risk patients. Alteration of the nicorandil dose should be discussed with the appropriate specialist to optimise control of anginal symptoms in these patients.

## Abbreviations

CT: computed tomography; NAD: nicotinamide adenine dinucleotide; NADP: nicotinamide adenine diphosphate

## Consent

Written informed consent was obtained from the patient for publication of this case report. A copy of the written consent is available for review by the Editor-in-Chief of this journal.

## Competing interests

The authors declare that they have no competing interests.

## Authors' contributions

CG analysed and interpreted the patient data and SW conducted the literature review. Both CG and SW wrote the manuscript. The original idea was that of CB who supervised and edited the manuscript. All authors read and approved the final manuscript.
